# A Simplified Technique for Implant-Abutment Level Impression after Soft Tissue Adaptation around Provisional Restoration

**DOI:** 10.3390/dj4020014

**Published:** 2016-05-24

**Authors:** Ahmad Kutkut, Osama Abu-Hammad, Robert Frazer

**Affiliations:** 1 Division of Prosthodontics, Department of Oral health Practice, College of Dentistry, University of Kentucky, 800 Rose St. D646, Lexington, KY 40536, USA; 2 Faculty of Dentistry, Taibah University, Almadena Almunawara, Saudi Arabia; deoaah@gmail.com; 3 Division of Prosthodontics, Department of Oral health Practice, College of Dentistry, University of Kentucky, Lexington, KY, 40536, USA; rfrazer@uky.edu

**Keywords:** implant, abutment, impression, coping, analog, crown and bridge

## Abstract

Impression techniques for implant restorations can be implant level or abutment level impressions with open tray or closed tray techniques. Conventional implant-abutment level impression techniques are predictable for maximizing esthetic outcomes. Restoration of the implant traditionally requires the use of the metal or plastic impression copings, analogs, and laboratory components. Simplifying the dental implant restoration by reducing armamentarium through incorporating conventional techniques used daily for crowns and bridges will allow more general dentists to restore implants in their practices. The demonstrated technique is useful when modifications to implant abutments are required to correct the angulation of malpositioned implants. This technique utilizes conventional crown and bridge impression techniques. As an added benefit, it reduces costs by utilizing techniques used daily for crowns and bridges. The aim of this report is to describe a simplified conventional impression technique for custom abutments and modified prefabricated solid abutments for definitive restorations.

## 1. Introduction

Dental implants and implant restorations are preferable alternatives to conventional dentures and bridgeworks. New digital technology and enhanced biomaterials are simplifying the restoration of implants and making the chair side dental treatment quicker for patients [1]. The dental technology market is overwhelming and is experiencing unprecedented growth; sales of dental implants, abutments, and computer guided surgery are expected to exceed $1.54 billion by 2018 [2].

The implant component that serves to support and retain the prosthesis is referred to as the abutment. The abutment can be prefabricated or custom made. Custom abutments can be machined (milled) or cast to serve in those circumstances where prefabricated components are not feasible. Impression techniques for implant restorations can be implant level or abutment level, open tray or closed tray, and may use metal or plastic impression copings. Metal impression copings are more accurate than plastic copings [3]. The impression coping shape has more impact on impression inaccuracy than does the impression technique [4].

Peri-implant tissue remodeling is a continuous process occurring after surgical implant placement and through the restoration process. It has been documented in the literature that a biological width forms around the platform of implants at the time of restoration [5,6]. A bone loss of 1.5–2 mm occurs at the implant-abutment junction due to the presence of a micro-gap at the implant-abutment connection. This contaminates the implant platform and initiates inflammatory reactions and consequent bone resorption. Contamination is suggested to happen when the healing abutment is removed during placement of the impression coping and during definitive abutment placement. As a consequence of bone loss, 1 mm of soft tissue recession can generally be expected during the first year. Most of this loss occurs within the first three months following abutment connection surgery. Eighty percent of the recession was on the buccal side [5,6]. It is recommended to wait three months for the tissue to stabilize before either selecting a final abutment or making a final impression. As a general rule, one can anticipate approximately 1 mm of recession from the time of abutment connection surgery [5,6].

Kutkut *el al.* [7] reported a technique for reconstructing the implant emergence profile using titanium and zirconia custom implant abutments. To ensure an esthetic restoration, a provisional restoration should be fabricated on the definitive abutment to allow peri-implant soft tissue stability. Final modifications of the finish line on the definitive abutment and abutment level impression should be made after peri-implant soft tissue stability is achieved [7].

The aim of this report is to describe a simplified conventional impression technique for custom abutments and modified prefabricated solid abutments for definitive restorations. It is useful when modifications to prefabricated implant abutment are needed to correct the angulation of malpositioned implant [8,9].

## 2. Impression Technique

**Figure 1:**
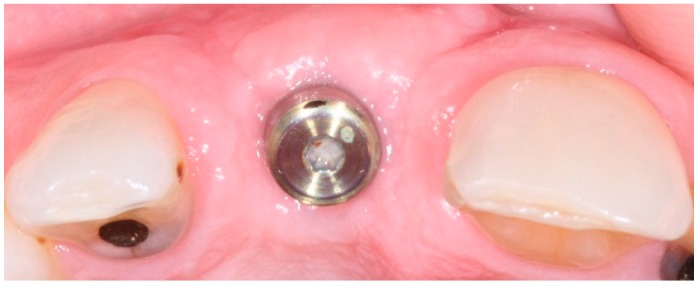
Healing abutment in place after three months of surgical implant placement.

**Figure 2:**
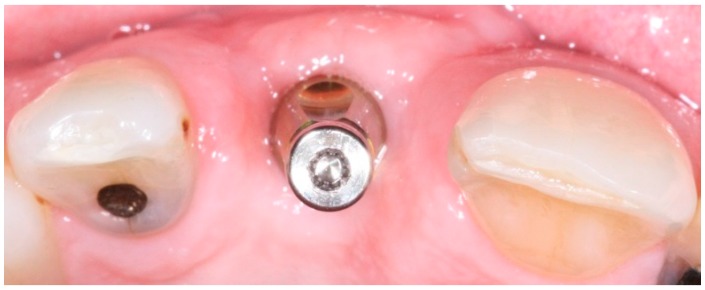
Implant level impression coping in place.

**Figure 3:**
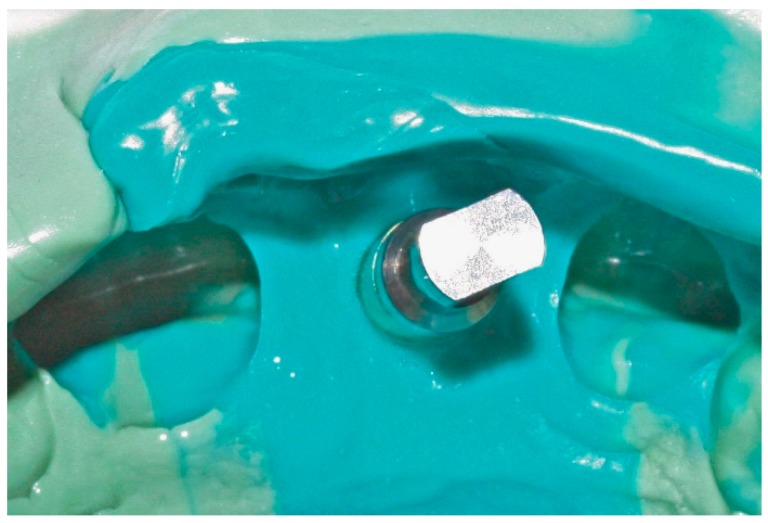
Implant replica connected into impression coping incorporated in PVS (polyvinyl siloxane) closed tray impression technique.

**Figure 4:**
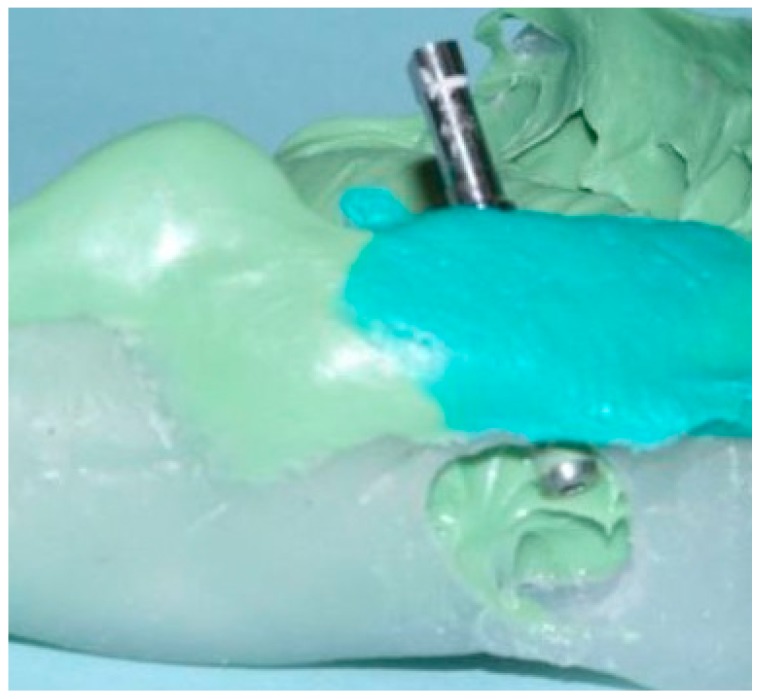
Implant replica connected into impression coping incorporated in PVS open tray impression technique due to malposition implant placement.

**Figure 5:**
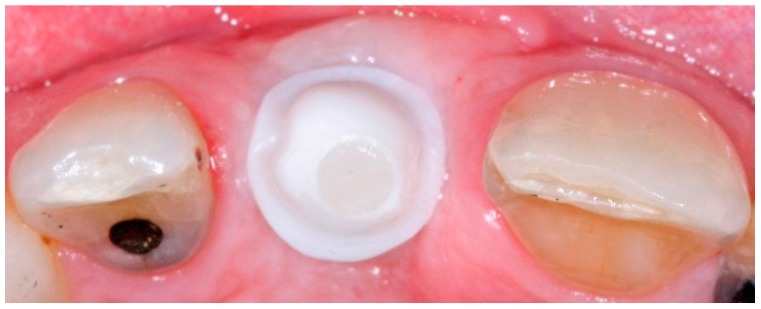
Zirconia custom abutment in place for anterior implant restoration with anatomic emergence profile.

**Figure 6:**
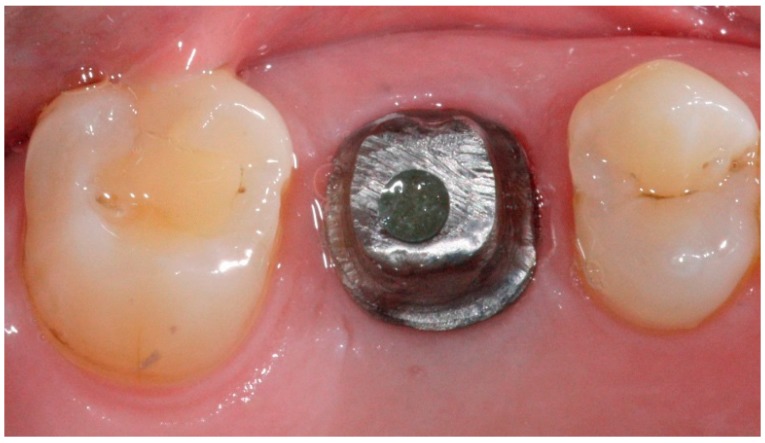
Titanium custom abutment in place for posterior implant restoration with anatomic emergence profile.

In conventional dental implant therapy, patients are asked to return for implant restoration after two or three months of healing following the surgical implant placement. In most cases, patients return with the healing abutment in place (Figure 1). After administrating appropriate topical and local anesthesia, the healing abutment is removed and the impression coping is screwed into the implant (Figure 2). The impression is made with a polyvinyl siloxane material (PVS; 3M ESPE, St. Paul, MN, USA) using open tray or closed tray impression techniques (Figure 3 and Figure 4) [10,11]. The impression is poured after the connection of the implant analogue in order to produce the working cast. In the laboratory, a prefabricated titanium abutment is modified or a titanium custom abutment is used for posterior implants whereas zirconia custom abutment is used for anterior implants [7]. The custom abutment is milled in the laboratory with an appropriate emergence profile (Figure 5 and Figure 6) [7,12,13,14,15].

**Figure 7:**
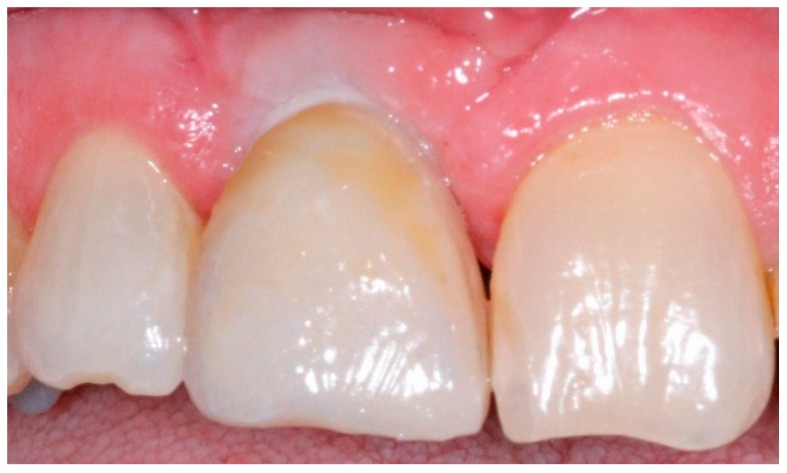
Provisional crown restoration for anterior implant.

**Figure 8:**
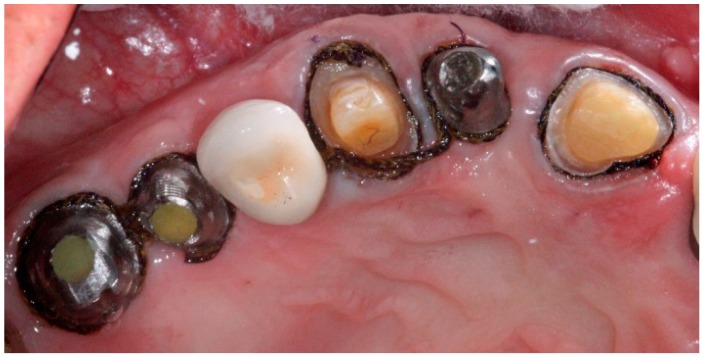
Abutment level impression as conventional crown and bridge impression technique.

**Figure 9:**
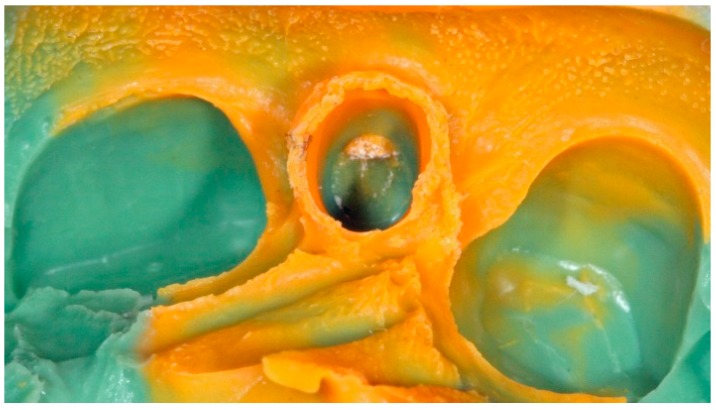
Conventional crown and bridge impression technique for implant abutment.

**Figure 10:**
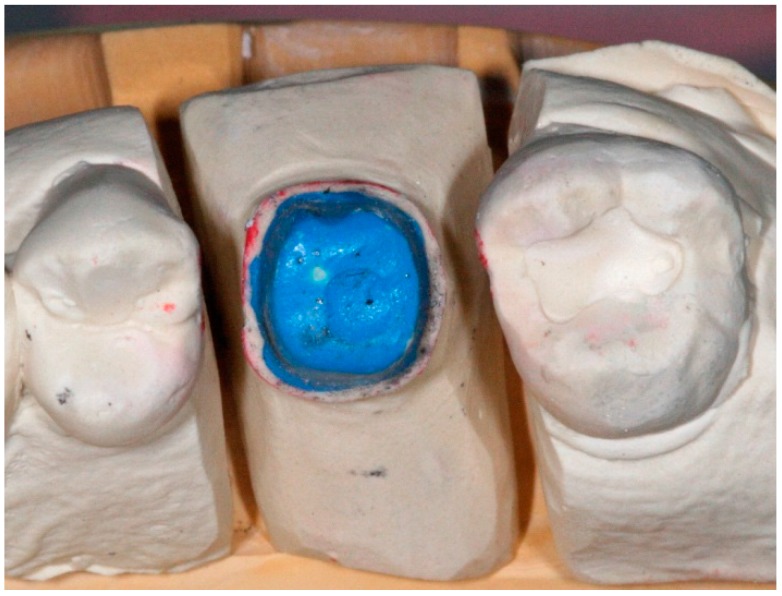
Conventional die for implant abutment supported crown fabrication.

Approximately two weeks after the implant level impression, the definitive abutment is screwed into the implant and evaluated for the finish line position. It should be approximately 1 mm below the gingival margin [5,6]. Any needed modifications are marked intraorally and the abutment is modified extraorally. The modified definitive abutment is polished and torqued to 35 Ncm. Screw access should be filled with Fermit™ (Patterson Dental, St. Paul, MN, USA). The provisional crown is relined with tooth colored acrylic resin over the new definitive abutment and cemented with temporary cement (Figure 7). All provisional crowns are placed in function with full contact in centric occlusion [7]. After one to three months of provisionalization, patients return for the definitive abutment level impression. The provisional crown is removed and any remaining cement is cleaned off the definitive abutment. After administration of an appropriate topical and local anesthesia, a single retraction cord (00′′) is packed around the abutment to retract just the peri-abutment soft tissue (Figure 8) [16]. After approximately five minutes, the retraction cord is removed and light body polyvinyl siloxane impression material is injected around the finish line of the implant abutment. Heavy body PVS impression material is placed in the tray and an impression is made using a closed tray impression technique. After complete polymerization of the impression material, the impression is retrieved and evaluated using the same criteria as for conventional crown and bridge impressions (Figure 9) [17]. The provisional crown is cemented back with temporary cement and excess cement is removed. The shade is selected, and an interocclusal record, facebow, and an impression of the opposing teeth are made and sent to the laboratory for conventional crown and bridge fabrication (Figure 10) [17].

**Figure 11:**
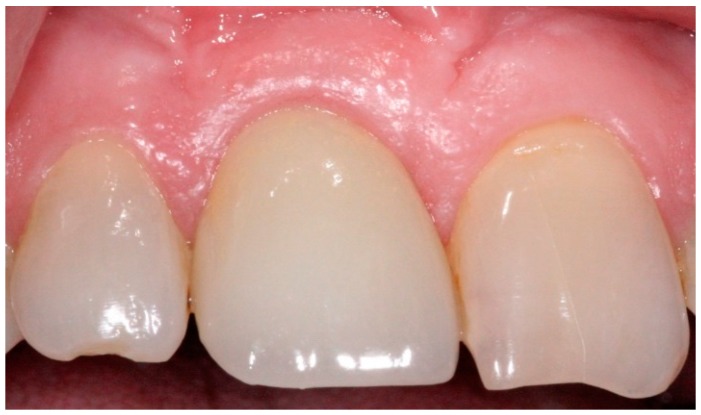
Anterior definitive crown cemented over definitive implant abutment.

**Figure 12:**
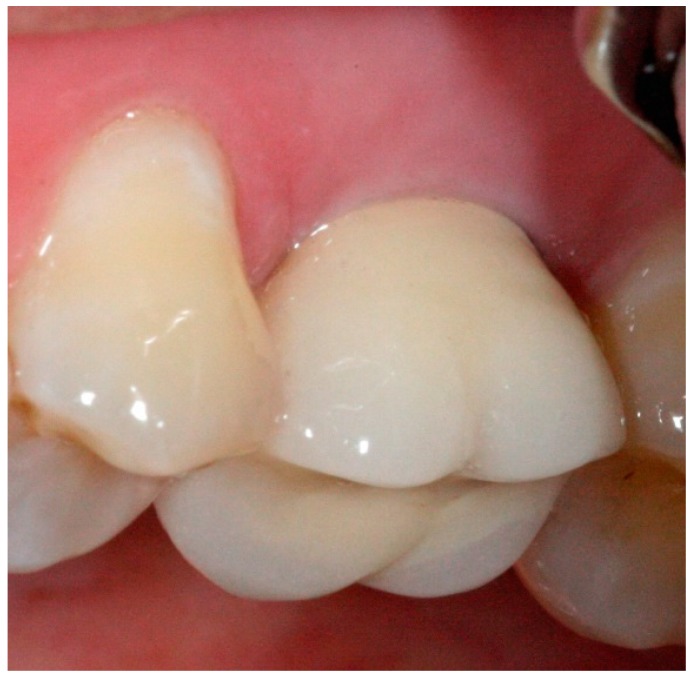
Posterior definitive crown cemented over definitive implant abutment.

The all ceramic or metal ceramic restorations are evaluated and the contacts and occlusion are adjusted as needed then cemented on the definitive abutments with permanent cement (Figure 11 and Figure 12). Occlusion is evaluated with an 8-μm foil (Shim stock Occlusion Foil, Patterson Dental, St. Paul, MN, USA) to achieve resistance to withdrawal only under maximal intercuspation [7,18]. All prosthetic restorations should utilize the manufacturer’s recommended components and protocol.

## 3. Discussion

Employing conventional crown and bridge impression techniques for accurate implant-abutment level impressions may be required when further modifications need to be applied to prefabricated or customized implant abutments [8,9]. It has been reported that the accuracy of the implant-abutment level impression is higher when the pick-up technique is used as opposed to conventional crown and bridge impression techniques [11,12].

Polyether and vinyl polysiloxane (VPS) have been recommended materials for the accurate implant-abutment level impressions [3,4,11].

The use of the conventional retraction cord technique or injectable materials to provide gingival retraction around implant abutments have been identified to be effective to expose finish lines and are suitable for conventional impression making methods [16].

There is a strong suggestion in literature that soft tissue around implant abutment connections can be sculpted through provisional restoration contours to optimize the esthetic outcomes [7,13]. Also, gold, titanium, and zirconia abutment materials exhibit excellent biological responses [13,14,15].

The complexity of restoring dental implants may require more armamentarium. Simplifying the restorative portion of implant supported restoration treatment through incorporating conventional crown and bridge impression techniques may allow more practitioners to restore dental implants in their practices. When this technique is utilized, special components (*i.e.*, impression caps, positioning cylinders) or laboratory parts (*i.e.*, multiple implant analogs) may not be required thereby reducing the costs and complexity of implant restorations and allowing the procedure to become more easily incorporated into any dental office.

## 4. Conclusions

- Peri-implant soft tissue stability around provisional restoration insures optimum esthetic outcomes.
- Employing well-known familiar impression techniques allow for the recording of optimum finish line positions after the appropriate adaptation of soft tissue around provisional implant restorations.
- This variation to the use of prefabricated impression copings allows the production of predictable restorations that are esthetically acceptable to the patient.
